# Exploring the link between visceral fat and cardiovascular disease in type 2 diabetes: evidence from ct measurements

**DOI:** 10.3389/fendo.2025.1635282

**Published:** 2025-10-02

**Authors:** Qing-Wu Wu, Yu-Hua He, Pei-Heng Li, Shi-Li Gu, Ran Song, Dong-Ying Zhang, Yun-Feng Zhu

**Affiliations:** ^1^ Department of Radiology, The First Affiliated Hospital of Xinxiang Medical University, Weihui, Xinxiang, Henan, China; ^2^ Department of Endocrinology, The First Affiliated Hospital of Xinxiang Medical University, Weihui, Xinxiang, Henan, China

**Keywords:** visceral fat area, type 2 diabetes, cardiovascular disease, physical examination data, quantitative computed tomography

## Abstract

**Background:**

Visceral fat is a well-established risk factor for cardiovascular disease (CVD) in patients with type 2 diabetes (T2DM). While visceral fat is recognized as a risk factor for CVD in T2DM patients, precise quantification of this relationship using direct CT measurements requires further validation in large populations. This study seeks to examine the cross-sectional association between VFA, as measured by CT, and prevalent CVD in T2DM patients, with the aim of informing risk management strategies in this group.

**Methods:**

This cross-sectional study analyzed data from 3,173 T2DM patients who underwent health screenings at Xinxiang First Affiliated Hospital between January 2020 and January 2025. CVD was defined as self-reported physician-diagnosed coronary artery disease, angina pectoris, stroke, congestive heart failure, or myocardial infarction, with verification through follow-up interviews when needed. CVD served as the dependent variable, while VFA, measured by CT, was the independent variable. VFA was categorized into quartiles. The association between VFA and CVD was assessed using univariate and multivariate analyses, smooth curve fitting with generalized additive models, and subgroup analyses.

**Results:**

The prevalence of CVD increased progressively across VFA quartiles in T2DM patients. After adjusting for confounders, VFA remained independently associated with prevalent CVD (OR = 1.43, 95% CI: 1.12 – 1.65, *P* < 0.001). Patients in the highest VFA quartile (Q4) had a 2.04-fold higher liver fat content compared to those in the lowest quartile (Q1) (95% CI: 1.56 – 2.94, *P* < 0.001). Subgroup analyses confirmed that this association was consistent across different populations (interaction *P* > 0.05).

**Conclusion:**

VFA is independently associated with prevalent CVD in T2DM patients. Future research should focus on the link between abdominal fat accumulation and CVD in this population.

## Introduction

Type 2 diabetes (T2DM) is becoming increasingly prevalent worldwide, with projections indicating that cases may rise to 1.31 billion by 2050 (1). China has one of the highest numbers of diabetes patients (2). T2DM is strongly linked to various cardiovascular diseases (CVD), and those with T2DM face a significantly higher risk of developing CVD, presenting a serious challenge to global healthcare systems (3). Recent studies underscore the importance of fat distribution, especially visceral fat, in the development of CVD in T2DM patients (4). Research has shown that visceral fat has a greater impact on metabolic health than subcutaneous fat. Excess visceral fat is associated with insulin resistance, dyslipidemia, and chronic inflammation, all of which are key risk factors for CVD (5, 6). Therefore, understanding the link between visceral fat and CVD in T2DM patients is vital for identifying high-risk individuals early and developing effective prevention strategies, which could significantly improve the quality of life for these patients.

Visceral fat area (VFA) has emerged as a significant marker of cardiovascular risk in T2DM patients (7, 8). Research indicates that visceral fat is metabolically active, releasing pro-inflammatory cytokines, adipokines, and free fatty acids into the portal circulation, which worsens insulin resistance and accelerates atherosclerosis (9–11). Unlike subcutaneous fat, which mainly stores energy, visceral fat contributes to disruptions in glucose and lipid metabolism, further increasing the risk of CVD in T2DM patients (12–14). Previous epidemiological and clinical studies, including large-scale imaging studies, have established the association between visceral adiposity and CVD in T2DM patients. However, many of these studies relied on indirect measures such as the visceral fat index, a composite measure that may be imprecise due to individual differences (15–17). Moreover, the visceral fat index may not be appropriate for Asian populations because of significant variations in fat distribution across ethnic groups (18). Other studies have used dual bioelectrical impedance analysis to estimate VFA (19–21), but this method lacks precision and cannot accurately distinguish between subcutaneous fat and VFA (22). In contrast, VFA measurement via chest CT images overcomes these limitations, providing a more precise assessment (23).

While the association between visceral adiposity and CVD in T2DM patients has been established, most evidence is based on indirect measurements or smaller study populations. This study aims to provide precise quantification of the VFA-CVD relationship using direct CT measurements in a large Chinese T2DM population. The objective is to assess the potential of VFA as a predictive biomarker for cardiovascular risk, which could inform more targeted interventions and ultimately improve cardiovascular outcomes in this high-risk population.

## Materials and methods

### Subjects and the inclusion criteria

This study protocol was approved by the Ethics Committee of Xinxiang First Affiliated Hospital (approval number: EC-025-374). The requirement for individual informed consent was waived by the Ethics Committee due to the retrospective design and strict anonymization of all patient data, in accordance with the Declaration of Helsinki and the Council for International Organizations of Medical Sciences (CIOMS) International Ethical Guidelines for Biomedical Research Involving Human Subjects. All data collection and processing strictly adhered to institutional data protection protocols, with researchers having access only to de-identified data. This study was conducted and reported following the Strengthening the Reporting of Observational Studies in Epidemiology (STROBE) guidelines. All research personnel completed medical ethics training and signed confidentiality agreements.

A retrospective analysis was performed on the medical records of adult T2DM patients who underwent health examinations at Xinxiang First Affiliated Hospital between January 2020 and January 2025. The inclusion criteria were: (1) T2DM patients who had a low-dose chest CT scan assessing VFA; (2) aged 20 to 80 years; and (3) complete demographic and questionnaire data. Exclusion criteria were patients with a historical or current diagnosis of any cancer, severe liver or kidney disease, thyroid disease, recent significant weight fluctuations (≥5%), pregnancy or breastfeeding, mental disturbance, or extreme values in examination results.

**Figure 1:**
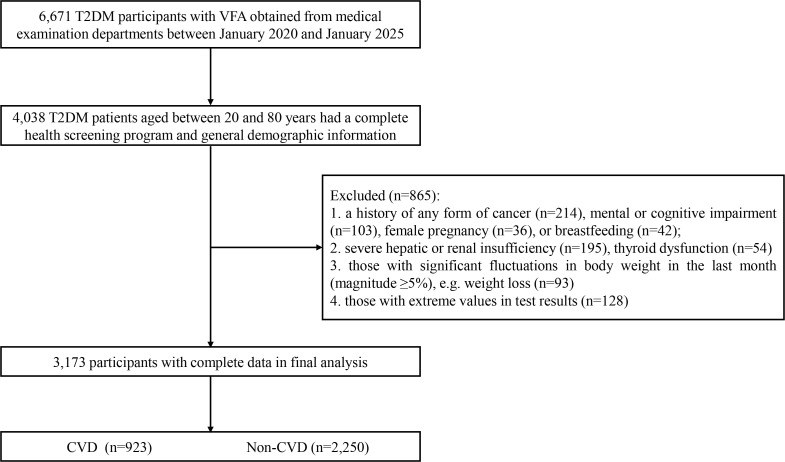
Flowchart of participants selection.

Initially, 6,671 T2DM patients were enrolled. After applying the exclusion criteria, 3,173 patients were retained for analysis, with 865 excluded. Among the retained patients, 923 (29.1%) reported a history of physician-diagnosed CVD (verified through our quality control procedures) and 2,250 (70.9%) reported no history of CVD. General demographic information, medical history, and medication history were collected through face-to-face interviews conducted by trained researchers. A detailed flowchart of the case selection process is shown in **Figure 1**.

### Definitions of variables

The independent variable was VFA in T2DM, measured by quantitative CT (QCT). VFA was defined as the intraperitoneal fatty area enclosed by the peritoneal wall or fascia transversalis muscle at the level of the L2/3 intervertebral space and the umbilicus, measured in square centimeters (cm²). CVD served as the dependent variable in this study. CVD was defined as a composite endpoint including any of the following physician-diagnosed conditions: coronary artery disease, angina pectoris, stroke, congestive heart failure, or myocardial infarction. CVD diagnosis was determined based on patient self-report of previous physician diagnosis obtained through standardized face-to-face interviews using a structured medical questionnaire. During the interview, trained researchers specifically asked each T2DM patient: ‘Has a doctor or healthcare professional ever diagnosed you with any of the following cardiovascular conditions: (1) coronary artery disease, (2) angina pectoris, (3) stroke, (4) congestive heart failure, or (5) myocardial infarction?’ A positive response to any of these five conditions was classified as having CVD. To ensure data quality and accuracy, any discrepancies or missing information regarding CVD history were systematically reconfirmed with participants through additional in-person interviews or telephone follow-up. These physician-diagnosed cardiovascular disease histories were classified according to the International Statistical Classification of Diseases and Related Health Problems, 10th edition (ICD-10), using the following specific codes: coronary artery disease (I20-I25.9), angina pectoris (I20.0-I20.9), congestive heart failure (I50.0, I50.1, I50.9), myocardial infarction (I21-I23), and stroke (I60-I69) (24). All analyses were based on verified CVD status as defined above, with quality control measures implemented to ensure data accuracy.

The diagnosis of T2DM followed the American Diabetes Association criteria (25): a previous physician diagnosis of diabetes, current treatment with hypoglycemic medications, fasting plasma glucose (FPG) ≥ 7.0 mmol/L, glycosylated hemoglobin (HbA1c) level ≥ 6.5%, 2-hour oral glucose tolerance test (OGTT) blood glucose ≥ 11.1 mmol/L, or use of insulin or oral hypoglycemic agents.

Body mass index (BMI) was calculated as weight (kg)/height² (m²) and categorized according to Chinese standards (26): normal weight (< 24 kg/m²), overweight (≥ 24 kg/m², < 28 kg/m²), and obese (≥ 28 kg/m²).

Hypertension was defined as having two consecutive measurements of systolic blood pressure (SBP) ≥ 140 mmHg or diastolic blood pressure (DBP) ≥ 90 mmHg, self-reported hypertension, use of antihypertensive drugs, or current antihypertensive therapy (27).

Estimated glomerular filtration rate (eGFR) was calculated using the formula: eGFR = 175 * serum creatinine^ (-1.154) * age^ (-0.203) * 0.742 (if female) * 1.212 (if black) (28). eGFR is expressed in mL/min/1.73 m², with serum creatinine in mg/dL and age in years.

Current smoking was defined as self-reported smoking by the participant. Current alcohol consumption was defined as the intake of at least one alcoholic beverage per week in the 12 months preceding the health screening.

All T2DM patients were divided into quartiles based on VFA levels: Q1 (33.30–172.00 cm²), Q2 (172.10–219.90 cm²), Q3 (220.00–271.90 cm²), and Q4 (272.10–497.90 cm²).

### Laboratory measurements

All researchers underwent standardized training to ensure objectivity and accuracy. Before the examination, they collected basic information from participants using a standardized questionnaire. This included a history of cardiovascular disease, liver and kidney disease, various cancers, and the use of diabetes, hypertension, and lipid-lowering medications, as well as recent weight changes. After completing the questionnaires, the data was organized, summarized, and verified. Any discrepancies or missing information, particularly regarding cardiovascular disease history, were systematically reconfirmed with participants through additional in-person interviews or telephone follow-up to ensure data accuracy and completeness.

Height was measured using a stadiometer, and weight was recorded with a weighing scale. Fasting venous blood samples were collected from all participants at 8 a.m. after a 12-hour fast. These samples were analyzed for creatinine (Cre), blood urea nitrogen (BUN), total cholesterol (TC), low-density lipoprotein cholesterol (LDL-C), triglycerides (TG), high-density lipoprotein cholesterol (HDL-C), FBG, and HbA1c. FBG was measured using an Olympus^®^ AU 5800 fully automated biochemistry analyzer (Beckman Coulter Inc., Brea, CA, USA). Other biochemical parameters were measured following standard laboratory protocols. Blood pressure (SBP and DBP) was measured using an electronic sphygmomanometer (OMRON U30, Omron Corporation, Kyoto, Japan). Measurements were taken on the right arm of each T2DM patient, positioned semi-flexed at heart level.

### VFA measurement

**Figure 2:**
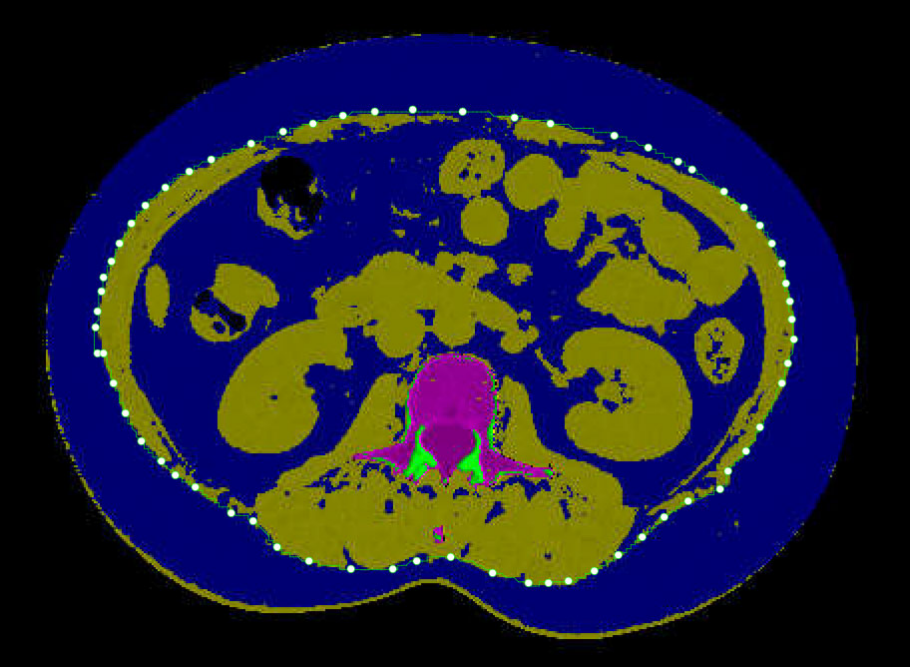
Abdominal quantitative computed tomography (QCT) image demonstrates abdominal fat distribution. The dark blue area represents non-adipose tissue and background, yellow regions indicate subcutaneous and visceral fat, and the magenta area represents specific visceral structures. The white dotted line delineates the boundary between subcutaneous and visceral fat compartments.

VFA was measured using low-dose chest CT scan data, a routine examination for assessing pulmonary lesions during health check-ups. The scan range included the L3 vertebra, minimizing unnecessary radiation exposure. All participants were scanned using the same 64-detector row CT scanner with standardized parameters: slice thickness 5.0 mm, tube voltage 120 kVp, tube current 100–150 mAs (auto-adjusted based on patient habitus), reconstruction matrix 512×512, calibrated weekly with a phantom (Mindways, Austin, TX, USA) to ensure consistent data quality. After scanning, a trained radiologist measured VFA using the QCT Pro 6.1 supplemental tissue measurement application from Mindways Software. This software performs QCT measurements on two standardized anatomical levels: (1) L2/3 intervertebral space, identified using sagittal reformatted images; (2) umbilicus level, verified by external anatomical landmarks and coronal reformats based on chest CT scan data. The application automatically segmented adipose tissue using standardized Hounsfield unit thresholds (-190 to -30 HU) and calculated the VFA in these slices. The final VFA value represents the average of measurements from both anatomical levels. To minimize measurement errors, care was taken to avoid artifacts from lumbar internal fixation, intestinal gas, or high-density contents. As illustrated in **Figure 2**. This measurement technique has been validated in the Chinese population (29). Further details on the measurement process can be found in previous studies (30, 31).

To ensure measurement reliability, intra-observer reproducibility was assessed by repeating measurements on 300 randomly selected cases (10% of total sample) after a 4-week interval, achieving an intraclass correlation coefficient (ICC) of 0.96 (95% CI: 0.94-0.98).

### Statistical analysis

All statistical analyses were conducted using R version 4.2.0 (R Foundation) and EmpowerStats (http://www.empowerstats.com, X&Y Solutions, Inc., Boston, MA). All tests were two-tailed, with a significance level set at *P* < 0.05.

Normality tests were performed on all datasets to assess continuous variables. Normally distributed continuous variables were reported as mean ± standard deviation, and group differences were evaluated using t-tests or rank-sum tests. Categorical variables were presented as frequencies and percentages, with comparisons made using chi-square tests.

Univariate analysis was used to evaluate the impact of various variables on CVD. Subsequently, multivariate logistic regression was conducted to assess the relationship between VFA and CVD, adjusting for covariates including sex, age, ethnicity, hypertensive medication, diabetes medication, lipid-lowering drugs, current smoking, current alcohol consumption, hypertension, BMI, BUN, creatinine, eGFR, LDL-C, TG, and HDL-C. Covariates with a variance inflation factor (VIF) >10 were excluded. Four models were developed in this study: a crude model with no adjustments, Model I adjust for demographic variables (sex, age, and ethnicity), Model II adjusting for demographic factors along with hypertensive drugs, diabetes drugs, lipid-lowering drugs, current smoking, alcohol consumption, hypertension, and BMI, and Model III adjusting for all previously mentioned confounders. The results from Model III were used for subsequent analyses. VFA was categorized into quartiles, with the lowest quartile serving as the reference group, to assess the relationship between VFA and CVD. A generalized additive model (GAM) with smooth curve fitting was employed to explore the dose-response relationship between VFA and CVD. Finally, stratified analyses and interaction tests based on Model III were performed to determine whether the relationship between VFA and CVD was consistent across different subgroups.

## Results

### Baseline details about T2DM

**Figure 3:**
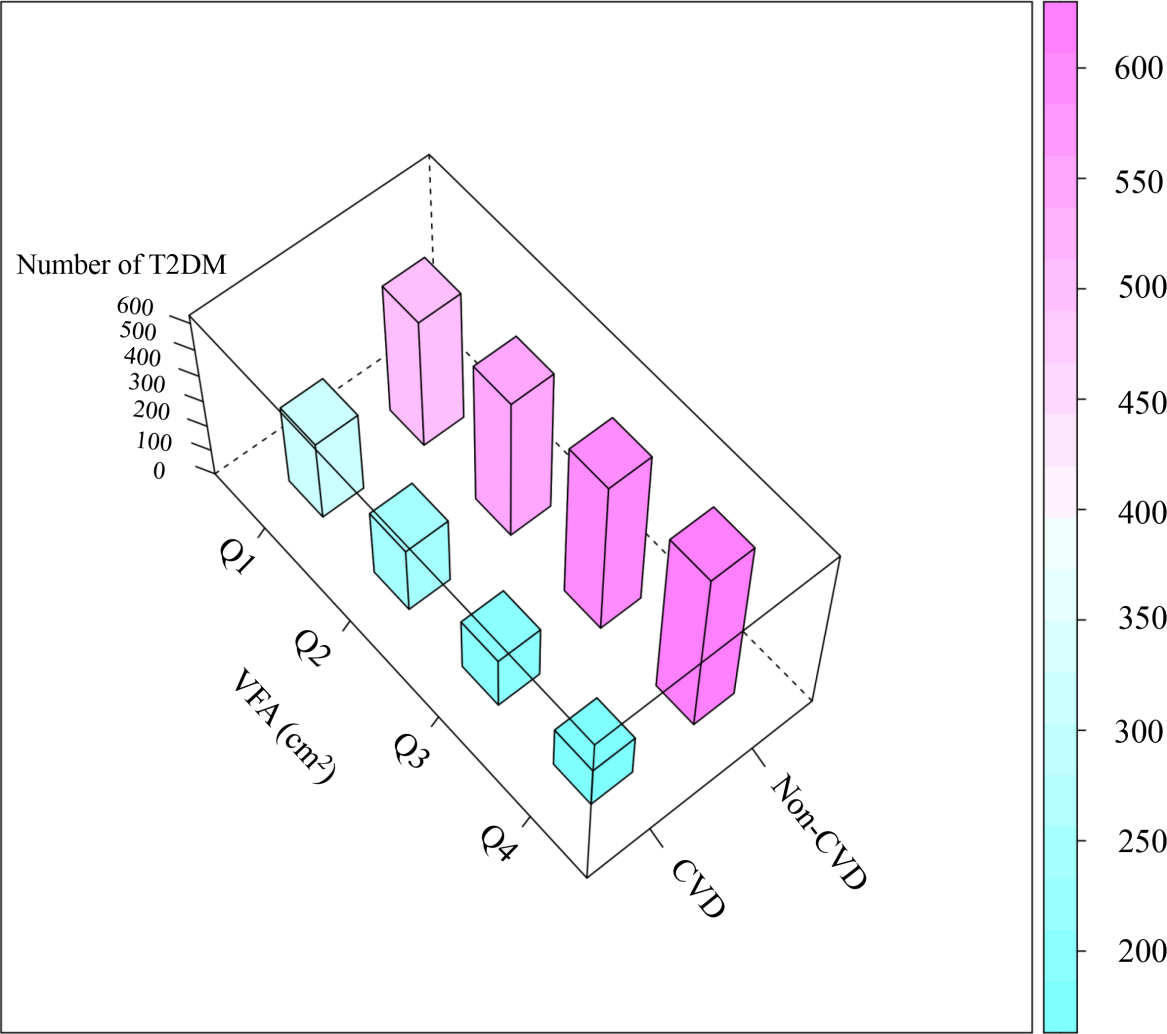
The total height of each quartile column of the 3D histogram of CVD incidence by quartile of VFA represents the total number of people in that group. VFA, visceral fat area; CVD, cardiovascular disease.

This study included 3,173 T2DM participants, comprising 2,254 men and 919 women. Participants were divided into four groups based on visceral fat area (VFA) quartiles: Q1 (33.30–172.00 cm², n = 793), Q2 (172.10–219.90 cm², n = 793), Q3 (220.00–271.90 cm², n = 792), and Q4 (272.10–497.90 cm², n = 795). As shown in **Figure 3**, the prevalence of CVD increased progressively across the VFA quartiles. Compared to the Q1 group, participants in the Q4 group (highest VFA) were more likely to be male, have a higher BMI, smoke and drink, have hypertension, use antihypertensive drugs, and have higher levels of Cre, TC, LDL-C, TG, FBG, and CVD prevalence (all *P* < 0.05). They also had lower HDL-C levels (*P* < 0.05). There were no significant differences in other variables (all *P* > 0.05), as shown in **Table 1**.

**Table 1:** Baseline characteristics of T2DM.

Variables	Q1 (33.30-172.00)	Q2 (172.10-219.90)	Q3 (220.00-271.90)	Q4 (272.10-497.90)	*P*-value
N	793	793	792	795	
Sex, n (%)					<0.001
Female	518 (65.32)	271 (34.17)	90 (11.36)	40 (5.03)	
Male	275 (34.68)	522 (65.83)	702 (88.64)	755 (94.97)	
Ethnic group, n (%)					0.638
Non-han	70 (8.83)	68 (8.58)	66 (8.33)	57 (7.17)	
Han	723 (91.17)	725 (91.42)	726 (91.67)	738 (92.83)	
Age, years	61.11 ± 11.62	61.05 ± 11.93	60.49 ± 11.70	60.30 ± 11.43	0.110
<60	382 (48.17)	400 (50.44)	413 (52.15)	430 (54.09)	
>=60	411 (51.83)	393 (49.56)	379 (47.85)	365 (45.91)	
BMI, kg/m^2^	23.10 ± 2.38	24.74 ± 2.46	25.80 ± 2.62	27.61 ± 3.24	<0.001
<24	561 (70.74)	345 (43.51)	197 (24.87)	112 (14.09)	
>=24, <28	199 (25.09)	371 (46.78)	448 (56.57)	325 (40.88)	
>=28	33 (4.16)	77 (9.71)	147 (18.56)	358 (45.03)	
Current smoking, n (%)					<0.001
No	759 (95.71)	768 (96.85)	736 (92.93)	730 (91.82)	
Yes	34 (4.29)	25 (3.15)	56 (7.07)	65 (8.18)	
Current drinking, n (%)					<0.001
No	761 (95.96)	742 (93.57)	725 (91.54)	714 (89.81)	
Yes	32 (4.04)	51 (6.43)	67 (8.46)	81 (10.19)	
Hypertension, n (%)					<0.001
No	498 (62.80)	489 (61.66)	436 (55.05)	396 (49.81)	
Yes	295 (37.20)	304 (38.34)	356 (44.95)	399 (50.19)	
Hypertensive drugs, n (%)					<0.001
No	580 (73.14)	564 (71.12)	517 (65.28)	488 (61.38)	
Yes	213 (26.86)	229 (28.88)	275 (34.72)	307 (38.62)	
Diabetes drugs, n (%)					0.416
No	753 (94.96)	760 (95.84)	763 (96.34)	754 (94.84)	
Yes	40 (5.04)	33 (4.16)	29 (3.66)	41 (5.16)	
Lipid-lowering drugs, n (%)					0.678
No	777 (97.98)	773 (97.48)	770 (97.22)	779 (97.99)	
Yes	16 (2.02)	20 (2.52)	22 (2.78)	16 (2.01)	
Cre, μmol/L	63.51 ± 19.43	68.98 ± 17.10	73.19 ± 19.55	75.26 ± 44.13	<0.001
BUN, mmol/L	5.07 ± 2.36	5.19 ± 2.25	5.31 ± 2.48	5.17 ± 2.42	0.252
eGFR, mL/min/1.73m^2^	97.19 ± 23.70	96.74 ± 22.11	97.81 ± 25.08	98.30 ± 25.01	0.584
TC, mmol/L	4.70 ± 1.15	4.75 ± 1.23	4.81 ± 1.23	4.82 ± 1.20	0.040
LDL-C, mmol/L	2.70 ± 0.92	2.72 ± 0.95	2.76 ± 0.94	2.89 ± 0.85	0.038
TG, mmol/L	1.65 ± 1.19	2.22 ± 2.22	2.47 ± 2.34	2.61 ± 2.26	<0.001
HDL-C, mmol/L	1.35 ± 0.34	1.20 ± 0.26	1.14 ± 0.26	1.12 ± 0.24	<0.001
FBG, mmol/L	7.07 ± 2.35	7.70 ± 2.41	7.62 ± 2.17	7.74 ± 2.23	<0.001
HbA1c, %	6.06 ± 2.78	6.13 ± 2.96	6.17 ± 2.82	6.08 ± 3.01	0.865
VFA, cm^2^	132.72 ± 29.71	196.75 ± 13.89	245.27 ± 14.76	320.49 ± 41.26	<0.001
CVD, n (%)					<0.001
No	630 (79.45)	591 (74.53)	538 (67.93)	491 (61.76)	
Yes	163 (20.55)	202 (25.47)	254 (32.07)	304 (38.24)	

BMI, body mass index; Cre, Creatinine; BUN, blood urea nitrogen; eGFR, estimated glomerular filtration rate; TC, total cholesterol; LDL-C, low-density lipoprotein cholesterol; TG, triglycerides; HDL-C, high-density lipoprotein cholesterol; FBG, fasting blood glucose; HbA1c, Glycosylated hemoglobin; VFA, visceral fat area; CVD, cardiovascular disease.

### Univariate analysis

Univariate logistic regression analysis was used to evaluate the impact of traditional variables on CVD and to select covariates for subsequent multivariate analysis. As shown in **Table 2**, male sex, older age, higher BMI, current smoking, current drinking, hypertension, eGFR, LDL-C, and TG were identified as risk factors for CVD (all *P* < 0.05). In contrast, the use of hypertensive drugs, diabetic drugs, lipid-lowering drugs, TC, and HDL-C levels were protective factors against CVD (all *P* < 0.05). Ethnic group, creatinine (Cre), and BUN did not show significant correlations (all *P* > 0.05).

**Table 2:** Univariate logistic regression analyses for CVD.

Variables	Statistics	OR (95%CI)	*P*-value
Sex, n (%)			
Female	919 (28.96)	Reference	
Male	2,254 (71.04)	1.19 (1.00, 1.42)	0.045
Ethnic group, n (%)			
Non-han	261 (8.23)	Reference	
Han	2,912 (91.77)	1.26 (0.94, 1.69)	0.121
Age, years			
<60	1,625 (51.21)	Reference	
>=60	1,548 (48.79)	1.31 (1.12, 1.52)	<0.001
BMI, kg/m^2^			
<24	1,215 (38.29)	Reference	
>=24, <28	1,343 (42.33)	1.46 (1.22, 1.74)	<0.001
>=28	615 (19.38)	1.90 (1.54, 2.35)	<0.001
Current smoking, n (%)			
No	2,993 (94.33)	Reference	
Yes	180 (5.67)	1.81 (1.33, 2.46)	<0.001
Current drinking, n (%)			
No	2,942 (92.72)	Reference	
Yes	231 (7.28)	1.78 (1.36, 2.35)	<0.001
Hypertension, n (%)			
No	1,819 (57.33)	Reference	
Yes	1,354 (42.67)	1.76 (1.51, 2.05)	<0.001
Hypertensive drugs, n (%)			
No	2,149 (67.73)	Reference	
Yes	1,024 (32.27)	-1.98 (-2.32, -1.69)	<0.001
Diabetic drugs, n (%)			
No	3,030 (95.49)	Reference	
Yes	143 (4.51)	-1.56 (-2.20, -1.10)	0.012
Lipid-lowering drugs, n (%)			
No	3,099 (97.67)	Reference	
Yes	74 (2.33)	-1.68 (-2.70, -1.05)	0.030
Cre, μmol/L	70.24 ± 27.75	1.00 (1.00, 1.01)	0.812
BUN, mmol/L	5.18 ± 2.38	1.01 (0.98, 1.04)	0.644
eGFR, mL/min/1.73m^2^	97.51 ± 24.00	1.00 (1.00, 1.01)	0.008
TC, mmol/L	4.77 ± 1.20	0.98 (0.92, 1.05)	0.598
LDL-C, mmol/L	2.72 ± 0.92	0.97 (0.89, 1.05)	0.024
TG, mmol/L	2.24 ± 2.09	1.04 (1.00, 1.08)	0.026
HDL-C, mmol/L	1.20 ± 0.29	-0.78 (-1.02, -0.60)	0.072
VFA, cm^2^	223.86 ± 73.92	1.00 (1.00, 1.01)	<0.001

BMI, body mass index; Cre, Creatinine; BUN, blood urea nitrogen; eGFR, estimated glomerular filtration rate; TC, total cholesterol; LDL-C, low-density lipoprotein cholesterol; TG, triglycerides; HDL-C, high-density lipoprotein cholesterol; FBG, fasting blood glucose; HbA1c, Glycosylated hemoglobin; VFA, visceral fat area; CVD, cardiovascular disease; OR, odd ratio.

### Associations between VFA and CVD of T2DM according to the different models

**Figure 4:**
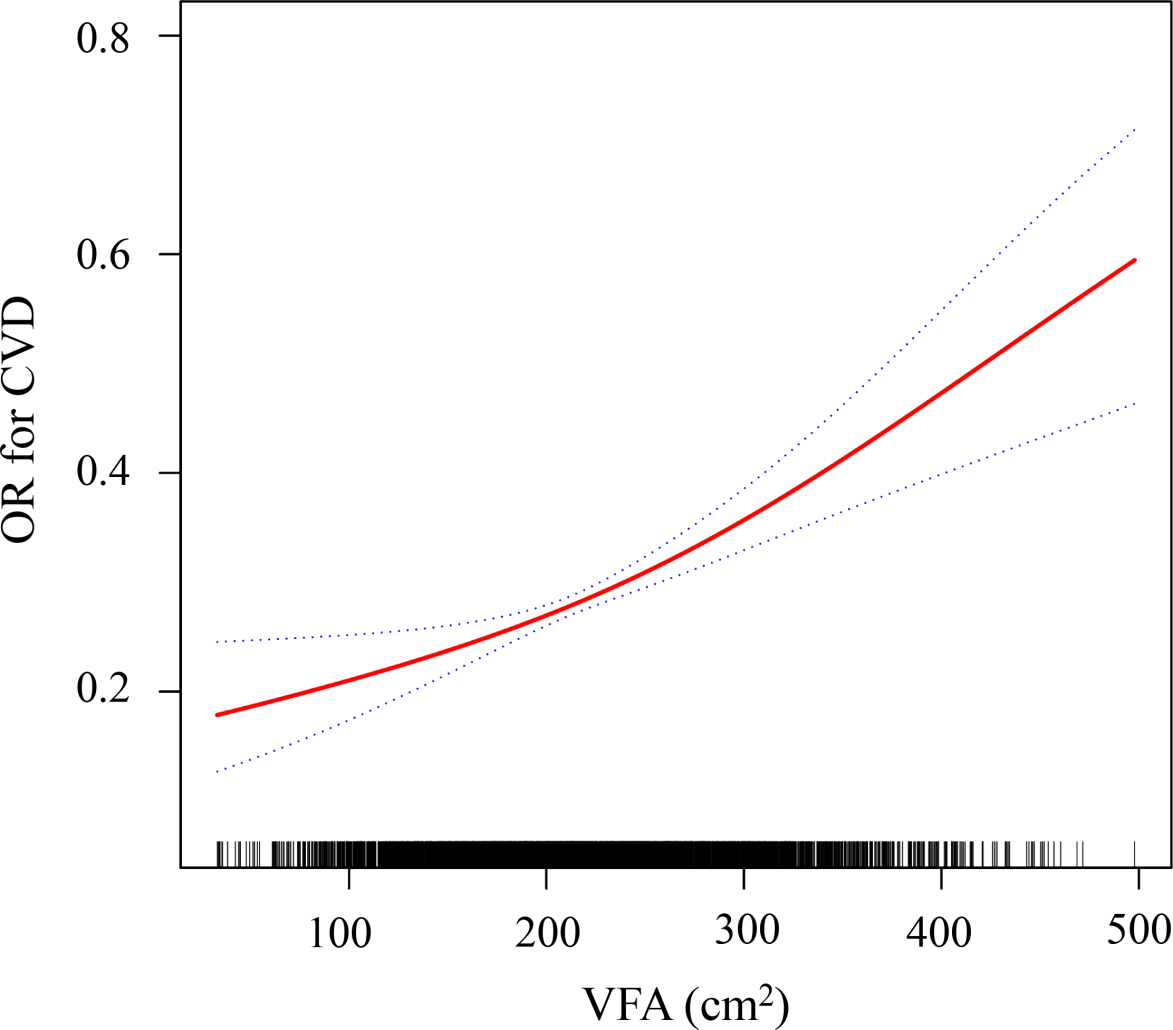
Generalized additive model (GAM) smoothing curve showing the linear dose–response association between VFA and CVD risk in T2DM (P_nonlinearity > 0.05). The red solid line represents the estimated probability, and the shaded area represents the 95% CI. All covariates were adjusted in this model. All covariates, including ex, age, ethnic group, hypertensive drugs, diabetic drugs, lipid-lowering drugs, current smoking, current drinking, hypertension, BMI, BUN, Cr, eGFR, LDL-C, TG, HDL-C were adjusted in this model. VFA, visceral fat area; CVD, cardiovascular disease; OR, odd ratio.

Multivariate regression analysis was conducted to account for confounding variables, and four models were developed. As shown in **Table 3**, the crude model, which did not adjust for any covariates, revealed a positive correlation between VFA and CVD (OR = 1.01, 95% CI: 1.00 - 1.02, *P* < 0.001). After adjusting for demographic variables such as sex, age, and ethnic group (Model I), a positive correlation between VFA and CVD was confirmed (OR = 1.02, 95% CI: 1.01– 1.06, *P* < 0.001). In Model II and Model III, VFA remained independently associated with an increased risk of CVD (OR = 1.43, 95% CI: 1.12 – 1.65, *P* < 0.001 in Model III). Specifically, for each unit increase in VFA, the odds of having prevalent CVD were 1.43 times higher. Additionally, when the VFA was divided into quartiles, after adjusting for confounding variables, the odds of having prevalent CVD in the Q4 group was 2.04 times higher than in the Q1 group (*P* < 0.001). Moreover, the association between VFA and CVD risk in T2DM patients was further examined through GAM smoothing curve fitting, which indicated a linear association (*P*-nonlinearity > 0.05). Each 10 cm² increase in VFA was associated with an odds ratio of 1.43 (95% CI: 1.12–1.65) for CVD risk (**Figure 4**).

**Table 3:** Multivariate regression analysis for prevalent CVD.

Variables	Crude model	Adjust I model	Adjust II model	Adjust III model
	OR (95%CI) *P-*value	OR (95%CI) *P-*value	OR (95%CI) *P-*value	OR (95%CI) *P-*value
VFA	1.01 (1.00, 1.02) <0.001	1.02 (1.01, 1.06) <0.001	1.21 (1.03, 1.36) <0.001	1.43 (1.12, 1.65) <0.001
VFA quartile				
Q1	Reference	Reference	Reference	Reference
Q2	1.62 (1.04, 1.67) 0.020	1.41 (1.10, 1.80) 0.006	1.31 (1.02, 1.70) 0.037	1.20 (1.00, 1.69) 0.045
Q3	1.82 (1.45, 2.29) <0.001	2.07 (1.60, 2.67) <0.001	1.98 (1.34, 2.35) <0.001	1.76 (1.32, 2.35) <0.001
Q4	2.39 (1.91, 2.99) <0.001	2.75 (2.13, 3.57) <0.001	2.18 (1.60, 2.97) <0.001	2.04 (1.56, 2.94) <0.001
*P* for trend	<0.001	<0.001	<0.001	<0.001

Non-adjusted model adjusts for: None.

Adjust I model adjust for: sex, age, and ethnic group.

Adjust II model adjust for: sex, age, ethnic group, hypertensive drugs, diabetic drugs, lipid-lowering drugs, current smoking, current drinking, hypertension, BMI.

Adjust III model adjust for: sex, age, ethnic group, hypertensive drugs, diabetic drugs, lipid-lowering drugs, current smoking, current drinking, hypertension, BMI, BUN, Cr, eGFR, LDL-C, TG, HDL-C. VFA, visceral fat area; CVD, cardiovascular disease; OR, odd ratio.

### Subgroup analysis

**Figure 5:**
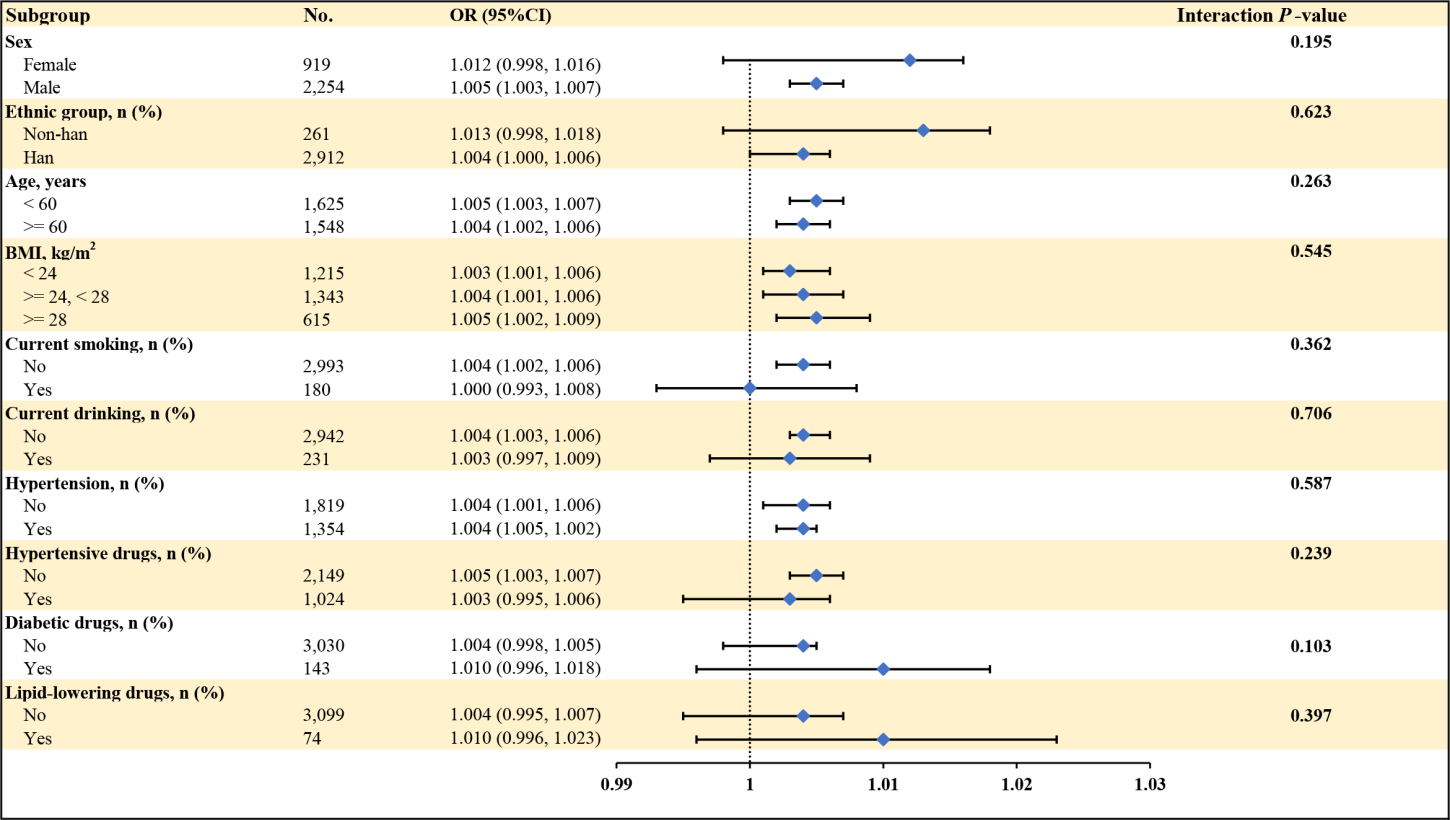
The association between VFA and the risk of CVD in T2DM according to different subgroups. Adjusted for all covariates except for this subgroup of variables. BMI, body mass index. VFA, visceral fat area; CVD, cardiovascular disease; OR, odd ratio.

As illustrated in **Figure 5**, the subgroup analyses revealed consistent findings. No significant interactions were observed when stratified by sex (female/male), ethnic group (non-Han/Han), age (<60 years/≥60 years), BMI (<24 kg/m²/≥24, <28 kg/m²/≥28 kg/m²), current smoking (yes/no), current drinking (yes/no), hypertension (yes/no), use of antihypertensive drugs (yes/no), diabetic drugs (yes/no), or lipid-lowering drugs (yes/no) (*P* for interaction > 0.05).

## Discussion

This cross-sectional analysis, conducted over six years of VFA data collection from health screening participants, demonstrated a positive association between VFA and prevalent CVD, even after adjusting for confounding variables. This relationship remained consistent across various subgroups, including sex, ethnic group, age, BMI, smoking status, drinking status, hypertension, and the use of antihypertensive, diabetic, and lipid-lowering drugs. This study provides important validation of the VFA-CVD relationship using precise QCT measurements in a large Chinese T2DM population. Our findings complement existing evidence by demonstrating this association through direct imaging-based quantification rather than indirect measures. These findings will aid primary care physicians in assessing CVD risk based on VFA and offer valuable guidance for the prevention and management of CVD in T2DM patients.

Our findings are consistent with several landmark studies that have established the relationship between visceral adiposity and CVD. Previous research using indirect measures has demonstrated this association, and our study extends these findings by providing precise quantification through direct CT measurements. Previous studies have shown that central obesity is a common feature of insulin resistance and its associated CVD (32–34). China has one of the highest prevalence rates of central obesity and T2DM (35–37). VFA is a key feature of central obesity (37). Excessive visceral fat accumulation can lead to the secretion of large amounts of inflammatory cytokines, resulting in low-grade inflammation, insulin resistance, and ultimately, the development of CVD (38). Moreover, inflamed adipocytes significantly reduce the production and secretion of adiponectin, impairing its role in protecting pancreatic β-cells from lipotoxicity and enhancing insulin sensitivity (39). While earlier research has suggested that VFA is one of the most valuable predictors of CVD in T2DM patients (20, 21, 40), the precise relationship between accurately measured VFA and CVD risk remains unclear. A recent multicenter study found that even T2DM patients with visceral obesity, but normal BMI have a significantly increased risk of developing atherosclerotic CVD (4). These findings highlight the importance of assessing VFA in T2DM patients. Therefore, we believe that precise VFA measurement should be prioritized over other indicators when evaluating CVD risk in T2DM patients. In this study, we used low-dose chest CT to measure VFA, with data derived from chest CT scans originally performed for lung cancer screening in T2DM patients. This approach ensures accurate VFA measurement while minimizing additional radiation exposure from repeated scans.

Building on this foundation, our study assessed the relationship between VFA and CVD risk by analyzing health examination data from T2DM patients. We observed that CVD incidence increased progressively with higher VFA levels. Even after adjusting for confounding factors, VFA remained independently and positively associated with prevalent CVD across multiple regression models. Importantly, this relationship remained robust across various subgroup analyses, consistent with previous findings using VFA measured by dual bioelectrical impedance analysis, which also suggested that VFA could be a risk marker for CVD in T2DM patients (20, 21, 41). Visceral adipose tissue, primarily located in the mesentery and omentum, drains directly into the liver through the portal circulation. Compared to subcutaneous adipocytes, visceral adipocytes are more metabolically active, more sensitive to lipolysis, and more resistant to insulin (12). Additionally, increased VFA promotes the secretion of plasminogen activator inhibitor-1 (PAI-1), leading to a hypercoagulable state, thereby raising the risk of atherosclerosis and coronary heart disease (42, 43). A prospective study of 97 hemodialysis patients reported that VFA is an independent predictor of CVD and all-cause mortality in this population (44). Another study involving 15,350 adult hypertensive participants found that visceral fat accumulation and prolonged exposure to high levels of visceral fat are significant risk factors for CVD (45). These findings underscore the robustness of VFA as a predictor of CVD events across various populations.

However, a recent longitudinal study based on the Chinese adult population identified a nonlinear relationship between the Chinese Visceral Adiposity Index (CVAI) and coronary heart disease risk, with a stronger association in men than in women (16). Unlike our study, this research used CVAI as the dependent variable, which includes waist circumference, BMI, triglycerides, HDL-C, and age (46). Although CVAI may better estimate visceral fat content in Chinese individuals, we believe the study may not have sufficiently accounted for confounding factors, potentially explaining the discrepancy with our findings. Additionally, a recent prospective study of 704 adult T2DM patients reported that perirenal fat thickness is an independent predictor of 10-year CVD risk in T2DM patients (47). This suggests that future research should explore the relationship between various visceral fat deposits and CVD in T2DM patients, allowing for the development of more precise prevention strategies to reduce CVD risk.

## Limitations and strengths

The key strengths of this study include: First, VFA was measured using QCT, enabling precise measurements based on lung cancer screening CT data without additional radiation exposure. Second, rigorous statistical methods were employed, offering robust support for the adjusted logistic regression analyses. However, the study has important limitations. Most importantly, the cross-sectional design of this study prevents establishment of causal relationships between VFA and CVD. Our findings demonstrate statistical association only and cannot determine whether VFA accumulation leads to CVD development, whether underlying CVD contributes to visceral fat accumulation, or whether both are consequences of common underlying pathophysiological processes. The temporal sequence between VFA changes and CVD development cannot be determined from our data. Therefore, our results should be interpreted as evidence of cross-sectional association rather than causation, and longitudinal studies are needed to establish any potential causal relationships. Additionally, the health screening program’s constraints limit the collection of certain covariates, such as inflammatory markers (e.g., hs-CRP). Finally, the study was conducted at a single health screening center in China, which may limit the generalizability of the findings to other populations. What’s more, while our study provides precise quantification of the established VFA-CVD relationship, it should be acknowledged that this represents a validation and methodological refinement of existing knowledge rather than a novel discovery of previously unknown associations. Last, regarding CVD ascertainment, while our study relied on patient self-report of physician diagnoses, we implemented quality control measures including systematic verification of discrepancies or missing information through follow-up interviews. This approach, commonly used in large epidemiological studies, provides a balance between feasibility and accuracy. However, the lack of direct access to medical records means that some degree of recall bias or misclassification cannot be completely excluded, though our verification procedures likely minimized such errors.

## Conclusion

Our study validates and precisely quantifies the independent positive association between VFA and increased CVD risk in T2DM patients, confirming previous observations through direct imaging measurements, with this relationship remaining robust across various subgroups. These findings suggest that VFA could serve as a clinical marker for CVD risk in T2DM patients, emphasizing the importance of monitoring VFA changes as part of CVD prevention strategies. Future prospective longitudinal studies should focus on fat accumulation around different abdominal organs in T2DM patients and assess whether changes in fat deposits predict CVD development. Such investigations could be crucial for enhancing the quality of life for T2DM patients.

## Data Availability

The original contributions presented in the study are included in the article/supplementary material, further inquiries can be directed to the corresponding author/s.
